# Assessment of cold exposure-induced metabolic changes in mice using untargeted metabolomics

**DOI:** 10.3389/fmolb.2023.1228771

**Published:** 2023-08-31

**Authors:** Linqiang Gong, Shiyuan Zhao, Xue Chu, Hui Yang, Yanan Li, Shanshan Wei, Fengfeng Li, Yazhou Zhang, Shuhui Li, Pei Jiang

**Affiliations:** ^1^ Tengzhou Central People’s Hospital, Tengzhou, China; ^2^ Translational Pharmaceutical Laboratory, Jining First People’s Hospital, Shandong First Medical University, Jining, China; ^3^ Institute of Translational Pharmacy, Jining Medical Research Academy, Jining, China; ^4^ College of Marine Life Sciences, Ocean University of China, Qingdao, China; ^5^ Department of Pharmacy, Shandong Provincial Hospital Affiliated to Shandong First Medical University, Jinan, China; ^6^ Graduate Department, Shandong First Medical University (Shandong Academy of Medical Sciences), Jinan, China

**Keywords:** cold exposure, metabolite, gas chromatography-mass spectrometry, multivariate analysis, main tissues

## Abstract

**Background:** Cold exposure (CE) can effectively modulate adipose tissue metabolism and improve metabolic health. Although previous metabolomics studies have primarily focused on analyzing one or two samples from serum, brown adipose tissue (BAT), white adipose tissue (WAT), and liver samples, there is a significant lack of simultaneous analysis of multiple tissues regarding the metabolic changes induced by CE in mice. Therefore, our study aims to investigate the metabolic profiles of the major tissues involved.

**Methods:** A total of 14 male C57BL/6J mice were randomly assigned to two groups: the control group (*n* = 7) and the CE group (*n* = 7). Metabolite determination was carried out using gas chromatography-mass spectrometry (GC-MS), and multivariate analysis was employed to identify metabolites exhibiting differential expression between the two groups.

**Results:** In our study, we identified 32 discriminant metabolites in BAT, 17 in WAT, 21 in serum, 7 in the liver, 16 in the spleen, and 26 in the kidney, respectively. Among these metabolites, amino acids, fatty acids, and nucleotides emerged as the most significantly altered compounds. These metabolites were found to be associated with 12 differential metabolic pathways closely related to amino acids, fatty acids, and energy metabolism.

**Conclusion:** Our study may provide valuable insights into the metabolic effects induced by CE, and they have the potential to inspire novel approaches for treating metabolic diseases.

## 1 Introduction

There is a well-established relationship between ambient temperature and mortality rates (Vialard and Olivier, 2020; Burkart et al., 2021; Fatima et al., 2021). Cold temperatures, as a frequent fluctuation in ambient temperature, have a profound impact on human health. In cold environments, endothermic organisms rely on thermogenesis to maintain their core body temperature. This allows cells to carry out crucial physiological processes and functions (Nguyen et al., 2011).

Brown adipose tissue (BAT) is a unique type of tissue dedicated to the process of non-shivering thermogenesis (NST), mediated by uncoupling protein 1 (UCP1) (Nicholls et al., 1978; Ricquier, 2017). The activation of BAT can be induced by exposure to cold temperatures. Earlier rodent studies have indicated that age significantly influences heat production capabilities in response to cold, with older mice exhibiting a reduced thermogenic effect (Talan et al., 1985; Tatelman and Talan, 1990). Initially, researchers believed that BAT had limited thermogenic and metabolic functions in adult humans, due to the observation that BAT depots were more prevalent in infants (Lean, 1989). However, this perspective has recently been questioned as multiple independent studies have reported the presence of metabolically active BAT in healthy adult humans (Cypess et al., 2009; van Marken Lichtenbelt et al., 2009; Virtanen et al., 2009). Studies conducted on rodents and humans have demonstrated that cold-induced activation of BAT has several beneficial effects on metabolic health. These effects include enhancing glucose uptake (Virtanen et al., 2009; Orava et al., 2011), improving insulin sensitivity (Stanford et al., 2013; Hanssen et al., 2015), stimulating lipolysis (Zechner et al., 2012), reducing circulating levels of triglyceride (TAG) and cholesterol (Bartelt et al., 2011; Berbée et al., 2015; Iwen et al., 2017; Worthmann et al., 2017), as well as clearing circulating branched-chain amino acids (BCAAs) (Yoneshiro et al., 2019). Exposure to cold leads to a swift increase in fibroblast growth factor 21 (FGF21) expression in BAT (Chartoumpekis et al., 2011; Hondares et al., 2011; Fisher and Maratos-Flier, 2016). FGF21 plays a crucial role in promoting the expression of thermogenic genes, including *UCP1*, within BAT (Fisher et al., 2012). Acting as a systematic peptide hormone, FGF21 plays a significant role in regulating energy balance, as well as maintaining glucose and lipid homeostasis. Additionally, there is a strong correlation between FGF21 levels and decreased circulating levels of BCAAs (Jiang et al., 2015; Karusheva et al., 2019; Yu et al., 2021; Shah et al., 2023). These findings have reignited interest in increasing energy expenditure by CE and BAT activation to combat obesity and its associated metabolic complications, including diabetes, dyslipidemia, and cardiovascular diseases in adult humans (Kajimura and Saito, 2014; Becher et al., 2021).

However, several studies have indicated that the activation of BAT through exposure to cold may have negative effects on health, such as an elevated heart rate, increased blood pressure (Wang et al., 2022), and potential involvement in the progression of breast cancer (Singh et al., 2016). Given these concerns, it is essential to meticulously assess the implications of this approach. Consequently, it is crucial to thoroughly investigate the impact of CE on the functioning of various vital organs in the body. Currently, there is a notable absence of simultaneous analysis of multiple tissues regarding the metabolic changes induced by CE in mice.

Metabolomics, which involves the examination of small molecule metabolites in biological samples, offers a comprehensive understanding of samples and valuable insights into biological alterations resulting from disease or environmental interactions (Murphy and Sweedler, 2022). Techniques like gas chromatography-mass spectrometry (GC-MS) and liquid chromatography-mass spectrometry (LC-MS), have been extensively utilized for the analysis of numerous metabolites. GC-MS, known for its high sensitivity and capacity for high-throughput analysis, has emerged as a valuable tool in non-targeted metabolomics investigations (Papadimitropoulos et al., 2018).

Several studies have explored the metabolic alterations induced by CE using metabolomics. But thus far, these studies have primarily focused on analyzing one or two samples from serum, BAT, WAT, and liver (Lu et al., 2017; Hiroshima et al., 2018; Okamatsu-Ogura et al., 2020; Hou et al., 2021; Kovaničová et al., 2021; Chen et al., 2022). There has been a noticeable absence of simultaneous analysis of multiple tissues regarding the metabolic changes induced by CE. To the best of our knowledge, this present study represents the first metabolomics investigation simultaneously examining the effects of CE on multiple tissues, including serum, BAT, WAT, liver, spleen, and kidney. Our findings have the potential to provide fresh insights into the metabolic impacts induced by CE, and they may also present new ideas for treating metabolic diseases.

## 2 Materials and methods

### 2.1 Animal treatment

A total of 14 male C57BL/6J mice, aged 6 weeks, were obtained from Jinan Pengyue (Jinan, China). These mice were allowed unrestricted access to food and water for 1 week in a climate chamber set at an ambient temperature of 24°C ± 2°C, with a relative humidity of 40%, and a 12/12 h light/dark cycle. Subsequently, the mice were randomly assigned to two groups: the CE group (*n* = 7) and the control group (*n* = 7). The mice in the CE group were subjected to a temperature of 4°C for 4 h per day, continuously for two consecutive weeks. In contrast, the mice in the control group were maintained at room temperature (24°C ± 2°C) for the same duration. The body weight and food intake of the mice were recorded on a weekly basis. All experimental procedures were conducted in compliance with the Regulations of Experimental Animal Administration issued by the State Committee of Science and Technology of the People’s Republic of China and were approved by the University Ethics Committee (approval no. JNRM-2022-DW-054).

### 2.2 Sample collection and preparation

Twenty-four hours after the last CE session, the mice were humanely euthanized. Food was withheld before sample collection to ensure a 6-h fasting period for the mice. Anesthesia was administered through intraperitoneal injection of sodium pentobarbital at a dose of 50 mg/kg. Following enucleation of the eyeballs, blood samples were collected and subsequently centrifuged at 3,500 rpm for 8 min to obtain serum samples. For euthanasia, cervical dislocation was performed on all mice. Immediately afterward, each mouse was necropsied on an ice surface to acquire samples of interscapular BAT, inguinal WAT, liver, spleen and kidney. All tissue samples were washed with phosphate-buffered saline (PBS, pH 7.2), rapidly frozen in liquid nitrogen, and stored at −80°C in a refrigerator for future use.

To prepare the serum samples, 100 μL of serum was combined with 350 μL of methanol containing 100 μg/ml heptadecanoic acid. The solution was then vortexed and centrifuged at 14,000 rpm for 10 min at 4°C to collect the supernatant. The supernatant was transferred to a 2 mL tube and dried at 37°C under a flow of nitrogen gas. Subsequently, 80 μL of o-methylhydroxylamine hydrochloride (dissolved in pyridine at 15 mg/mL) was added to the dried sample and thoroughly mixed. The mixture was incubated at 70°C for 90 min. Next, 100 μL of N,O-bis (trimethylsilyl) trifluoroacetamide containing 1% trimethylchlorosilane (Sigma-Aldrich) was added to each sample, followed by a 60-min incubation at 70°C. The solution was then vortexed, centrifuged at 14,000 rpm for 2 min at 4°C, and filtered through a 0.22-μm filter membrane before GC-MS analysis. To prepare the tissue samples (BAT, WAT, liver, spleen, and kidney), 50 mg of each sample was homogenized in 1 mL of methanol containing 1 mg/mL heptadecanoic acid. After homogenization, the samples were centrifuged for 10 min at 20,913 × g at 4°C. The subsequent steps of the protocol were similar to those used for the serum samples. For the quality control samples (QCs), equal amounts of tissue samples from the control group and CE group were mixed together.

### 2.3 GC-MS analysis

The analysis of all samples was performed using a 7000°C mass spectrometer coupled with a 7890 B gas chromatograph system from Agilent Technologies (CA, United States). The separation of serum, BAT, WAT, liver, spleen and kidney samples was performed using an HP-5MS fused silica capillary column. Using helium gas as a carrier, a 1 µL aliquot of the derivative solution was processed in split mode (50:1), with the front inlet purge flow rate set to 3 mL/min and the gas flow rate set to 1 mL/min. The temperatures for the administration, transfer line, and ion source were maintained at 280°C, 250°C, and 230°C, respectively. The GC temperature program started at 60°C for 4 min, followed by an increase to 300°C at a rate of 8°C/min, and then held at 300°C for 5 min. For ionization, the voltage of the electron impact was set to −70 eV, and data acquisition occ1urred at a rate of 20 spectra per second. MS identification was performed using electrospray ionization (ESI) in full scan mode, with a mass-to-charge ratio (m/z) range of 50–800.

We have effectively uploaded the source data from our GC-MS analysis to MetaboLights. The identifier MTBLS8334 has been exclusively assigned to our research project. To access our study, simply follow this link: https://www.ebi.ac.uk/metabolights/MTBLS8334.

### 2.4 Multivariate statistical analysis

The initial analysis of the GC-MS data was conducted using Agilent Unknowns Analysis software and Mass Hunter Quantitative Analysis software from Agilent Technologies (United States). The SIMCA 14.1 software (Umetrics, Sweden) was employed for the statistical analysis of normalized peak area percentages. Orthogonal Projections to Latent Structures Discriminant Analysis (OPLS-DA) was performed to differentiate between the CE group and control group. OPLS-DA is a statistical method utilized for multivariate data analysis, especially when dealing with high-dimensional data and a limited number of samples. The primary objective of OPLS-DA is to identify significant differences and relationships between two or more groups or classes within a dataset (Bylesjö et al., 2006). The method achieves this by modeling the systematic variation between classes, such as different treatment groups or disease states, while effectively removing unrelated or confounding variations. Variable Importance in Projection (VIP) values estimate the importance of each variable in the projection used in a least squares regression model and is often used for variable selection (Mehmood et al., 2012; Pinto et al., 2012). A variable with a VIP value close to or greater than one can be considered important in given model. Variables with VIP values significantly less than one are less important and might be good candidates for exclusion from the model. Model validation was further verified using the permutation test (200 permutations). Two-tailed Student's t-tests were conducted using SPSS 19.0 software (SPSS, Chicago, IL, United States). Then *p* values obtained from the *t*-test were corrected using fdrtool (Strimmer, 2008). Metabolites with VIP values >1.0 in the OPLS-DA analysis and *p*-values adjusted <0.05 were considered statistically significant.

Differential endogenous metabolites were imported into MetaboAnalyst 5.0 (http://www.metaboanalyst.ca) as well as the Kyoto Encyclopedia of Genes and Genomes (KEGG; http://www.kegg.jp) for metabolic pathway analysis. Metabolic pathways with impact values >0 and *p*-values adjusted <0.05 were considered as significantly affected pathways.

## 3 Results

### 3.1 Impact of CE on food intake and body weight

**FIGURE 1:**
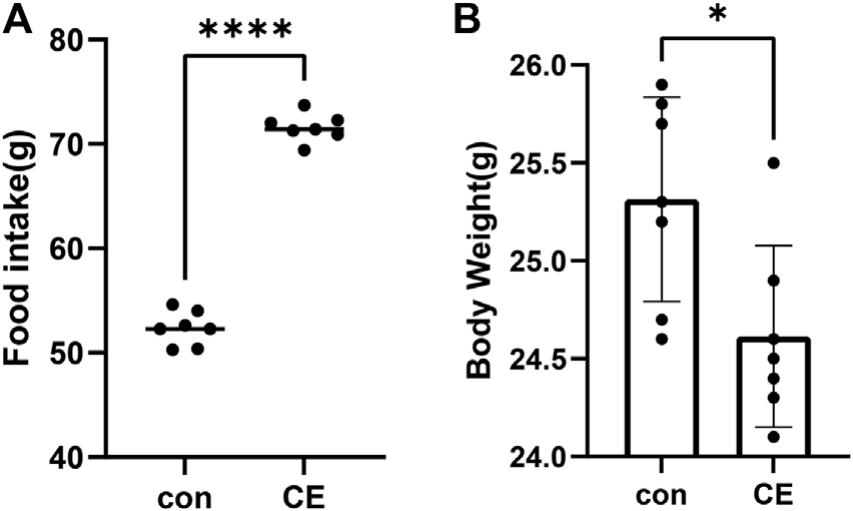
Impact of CE (cold exposure) on food intake and body weight. **(A)** Food intake during the entire 2-week period in CE and con (control) groups, **(B)** body weight over the full 2 weeks between these two groups. Data represent mean ± SEM, *n* = 7, ****p < 0.0001,*p < 0.05 compared to the control group.

The CE group exhibited a significant increase in food intake compared to the control group (*p*-values <0.0001, Figure 1A). Conversely, the CE group showed a decrease in body weight (*p*-values <0.05, Figure 1B). These findings, consistent with the previous report (Yang et al., 2017), suggest that CE induces increased energy expenditure.

### 3.2 GC-MS total ion chromatograms of samples

**FIGURE 2:**
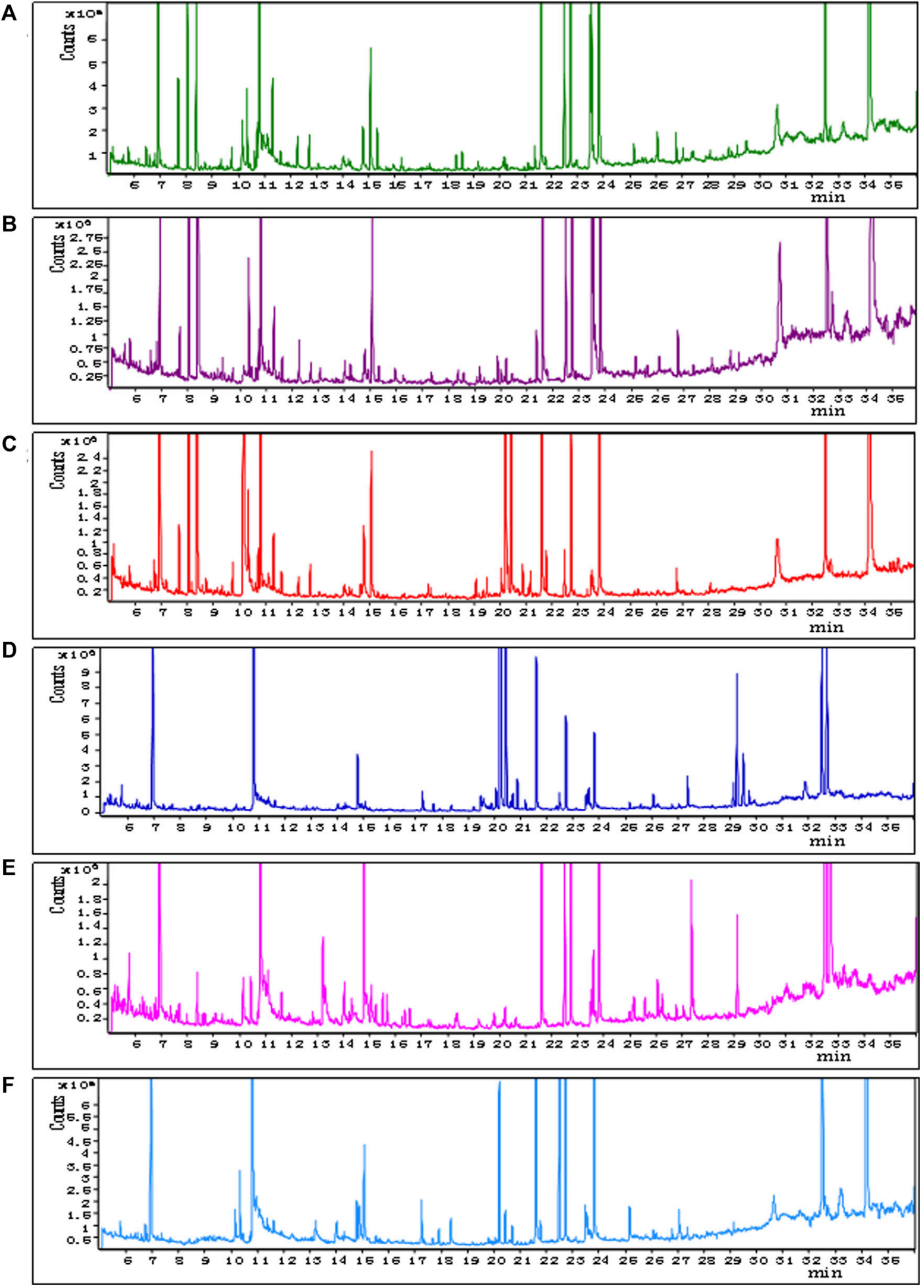
Representative GC–MS total ion current chromatograms from QCs. **(A)** BAT, **(B)** WAT, **(C)** serum, **(D)** liver, **(E)** spleen, **(F)** kidney (x-axis represents time and y-axis represents abundance).

The representative total ion chromatograms of QCs were shown in Figure 2; Details can be seen in Supplementary Material. The chromatograms demonstrate strong signal responses in all samples, indicating the detection of a diverse range of metabolites with a high peak capacity throughout the analysis. The retention time was consistent and the chromatograms of each tissue exhibited excellent reproducibility.

### 3.3 Multivariate statistical analysis

**FIGURE 3:**
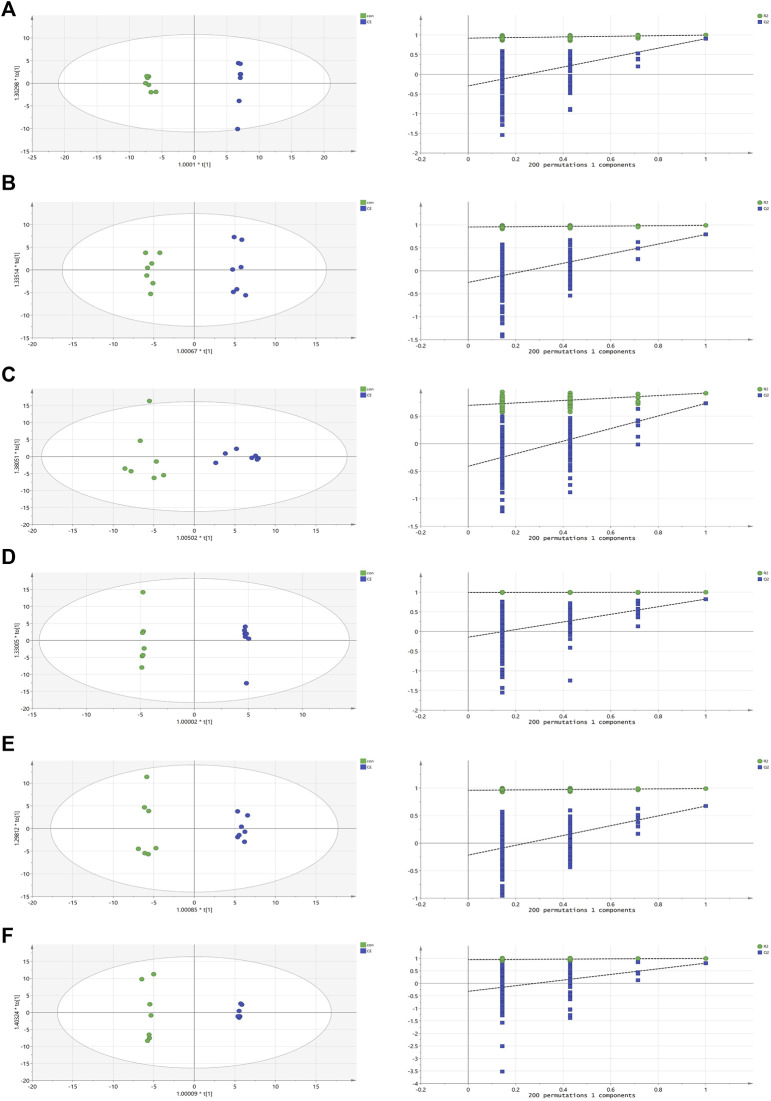
OPLS-DA scores and 200 permutation tests for OPLS-DA models. **(A)** BAT, **(B)** WAT, **(C)** serum, **(D)** liver, **(E)** spleen, **(F)** kidney. Statistical validation of the significant OPLS-DA models by permutation testing revealed no over-fitting (note that the blue regression line of the Q2 points intersect the vertical axis at values <0).

In OPLS-DA analysis, clear differences were observed between the CE group and the control group. The parameters (BAT R2X = 0.622, R2Y = 0.997, Q2 = 0.904; WAT R2X = 0.522, R2Y = 0.989, Q2 = 0.791; serum R2X = 0.59, R2Y = 0.918, Q2 = 0.731; liver R2X = 0.736, R2Y = 1, Q2 = 0.824; spleen R2X = 0.515, R2Y = 0.992, Q2 = 0.673; and kidney R2X = 0.669, R2Y = 0.997, Q2 = 0.805) indicated the effectiveness of the model, allowing clear differentiation between the CE group and the control group. Each parameter value was close to 1.0, signifying a stable and reliably predictive model. Model validation was verified through permutation tests (200 permutations), and the intersection of the blue regression lines (the Q2 points) and the vertical axis (on the left) all fell below zero (Figure 3).

**FIGURE 4:**
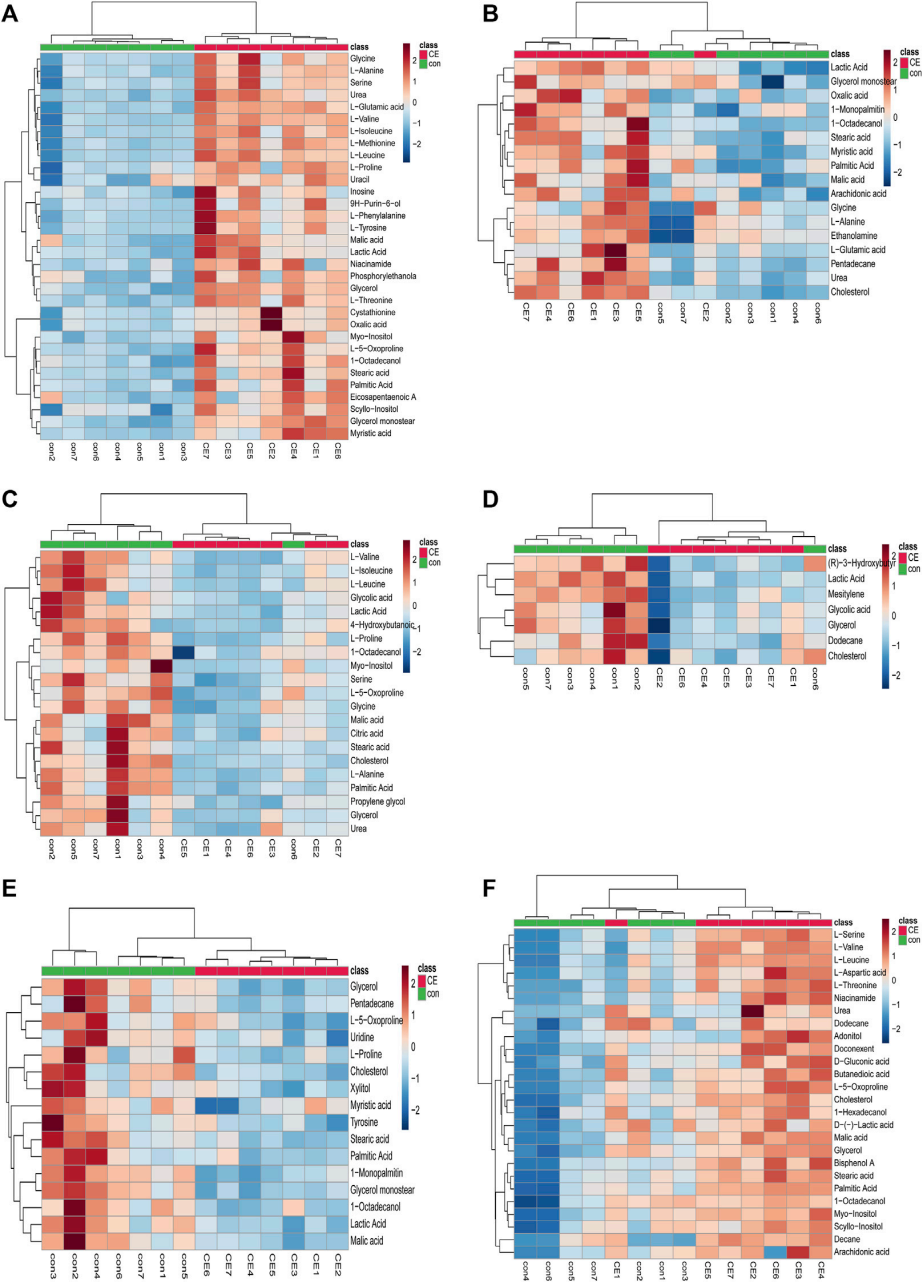
Heatmap of differential metabolites in CE and control groups. **(A)** BAT, **(B)** WAT, **(C)** serum, **(D)** liver, **(E)** spleen, **(F)** kidney. Red and blue represent upregulation and downregulation, respectively. Rows and columns correspond to metabolites and samples, respectively.

We used MetaboAnalyst 5.0 to investigate metabolic differences between the two groups. Cluster analysis of the expression of metabolites in tissues revealed that most samples were grouped into two differentiated clusters with only a small part of the sample cluster overlapping (Figure 4). These results were consistent with those of the OPLS-DA analysis.

### 3.4 Identification of discriminant metabolites

The significance threshold for identifying differential compounds between the control and CE groups was set at VIP values >1.0 and *p*-values adjusted <0.05. Metabolites meeting these criteria were considered significantly different. Furthermore, metabolites exhibiting an upward trend were indicated by fold change (FC) > 1, while a downward trend was indicated by FC < 1. There were 32, 17, 21, 7, 16 and 26 discriminant metabolites in BAT, WAT, serum, liver, spleen and kidney between the two groups, respectively. A detailed summary of altered metabolites in major tissues following exposure to cold was provided in Table 1.

**TABLE 1:** Differential metabolites detected in BAT, WAT, serum, liver, spleen and kidney between CE and control groups.

Metabolites	HMDB	VIP	P	P adjusted	Fold change
BAT					
L-Glutamic acid	HMDB0000148	1.43	1.73E-06	1.40E-05	3.07
Glycerol	HMDB0000131	1.41	2.60E-06	1.40E-05	1.87
L-Valine	HMDB0000883	1.42	2.91E-06	1.40E-05	2.68
Glycerol monostearate	HMDB0011535	1.40	6.60E-06	2.64E-05	1.84
L-Methionine	HMDB0000696	1.37	2.01E-05	6.60E-05	2.64
L-Leucine	HMDB0000687	1.34	3.97E-05	1.03E-04	2.79
L-Isoleucine	HMDB0000172	1.31	1.09E-04	2.15E-04	2.67
L-Threonine	HMDB0000167	1.35	1.89E-04	3.03E-04	2.12
L-Alanine	HMDB0000161	1.28	2.54E-04	3.80E-04	2.60
Urea	HMDB0000294	1.34	3.24E-04	4.51E-04	4.78
L-Phenylalanine	HMDB0000159	1.22	6.30E-04	7.03E-04	2.97
Serine	HMDB0062263	1.21	8.04E-04	8.14E-04	2.27
1-Octadecanol	HMDB0002350	1.21	8.52E-04	8.41E-04	1.42
L-5-Oxoproline	HMDB0000267	1.27	1.15E-03	9.82E-04	2.18
Myo-Inositol	HMDB0000211	1.13	2.17E-03	1.33E-03	1.52
Scyllo-Inositol	HMDB0006088	1.15	2.55E-03	1.45E-03	2.20
Stearic acid	HMDB0000827	1.12	2.68E-03	1.49E-03	1.82
Glycine	HMDB0000123	1.20	2.69E-03	1.49E-03	3.23
L-Proline	HMDB0000162	1.14	2.82E-03	1.53E-03	1.95
Myristic acid	HMDB0000806	1.23	3.03E-03	1.58E-03	1.95
Malic acid	HMDB0000744	1.11	3.17E-03	1.62E-03	2.30
Niacinamide	HMDB0001406	1.09	3.96E-03	1.93E-03	2.67
Lactic Acid	HMDB0000190	1.16	4.24E-03	2.04E-03	2.96
Uracil	HMDB0000300	1.10	4.33E-03	2.07E-03	1.66
Phosphorylethanolamine	HMDB0000224	1.08	5.80E-03	2.56E-03	1.76
L-Tyrosine	HMDB0000158	1.14	6.62E-03	2.80E-03	3.72
Inosine	HMDB0000195	1.05	6.92E-03	2.88E-03	2.47
Palmitic Acid	HMDB0000220	1.09	9.01E-03	3.41E-03	1.58
Cystathionine	HMDB0000099	1.03	9.13E-03	3.44E-03	2.46
Oxalic acid	HMDB0002329	1.03	9.21E-03	3.46E-03	2.42
Eicosapentaenoic Acid	HMDB0001999	1.01	9.99E-03	3.69E-03	2.00
9H-Purin-6-ol	HMDB0000157	1.04	1.60E-02	5.40E-03	4.40
WAT					
Urea	HMDB0000294	1.72	5.76E-05	1.90E-03	3.80
Cholesterol	HMDB0000067	1.69	1.42E-04	1.90E-03	1.72
Pentadecane	HMDB0059886	1.55	8.26E-04	4.68E-03	1.50
Myristic acid	HMDB0000806	1.45	2.98E-03	8.32E-03	1.64
L-Alanine	HMDB0000161	1.45	3.67E-03	8.82E-03	2.08
Lactic Acid	HMDB0000190	1.52	4.42E-03	9.23E-03	1.70
Oxalic acid	HMDB0002329	1.40	4.74E-03	9.36E-03	1.79
1-Octadecanol	HMDB0002350	1.49	5.93E-03	1.12E-02	1.48
Malic acid	HMDB0000744	1.35	9.73E-03	1.63E-02	1.81
Stearic acid	HMDB0000827	1.29	1.63E-02	2.27E-02	1.66
L-Glutamic acid	HMDB0000148	1.32	1.70E-02	2.32E-02	4.16
Arachidonic acid	HMDB0001043	1.21	2.40E-02	2.86E-02	1.91
Glycine	HMDB0000123	1.17	2.75E-02	3.09E-02	2.12
Glycerol monostearate	HMDB0011535	1.16	3.32E-02	3.42E-02	1.50
Ethanolamine	HMDB0000149	1.17	3.38E-02	3.45E-02	1.72
1-Monopalmitin	HMDB0011564	1.02	3.83E-02	3.76E-02	1.50
Palmitic Acid	HMDB0000220	1.08	4.96E-02	4.51E-02	1.46
Serum					
L-5-Oxoproline	HMDB0000267	1.55	7.95E-05	8.32E-04	0.56
L-Alanine	HMDB0000161	1.50	4.09E-04	1.84E-03	0.44
L-Proline	HMDB0000162	1.49	5.77E-04	2.01E-03	0.52
Serine	HMDB0062263	1.52	1.95E-03	3.41E-03	0.69
Glycine	HMDB0000123	1.42	2.95E-03	3.99E-03	0.69
Lactic Acid	HMDB0000190	1.33	3.24E-03	4.11E-03	0.50
Cholesterol	HMDB0000067	1.30	5.55E-03	4.71E-03	0.58
Malic acid	HMDB0000744	1.24	6.66E-03	4.88E-03	0.35
Palmitic Acid	HMDB0000220	1.31	6.75E-03	4.89E-03	0.54
L-Valine	HMDB0000883	1.38	6.86E-03	4.91E-03	0.62
Myo-Inositol	HMDB0000211	1.24	7.34E-03	4.96E-03	0.43
Propylene glycol	HMDB0001881	1.21	8.21E-03	5.15E-03	0.45
1-Octadecanol	HMDB0002350	1.24	1.13E-02	6.42E-03	0.63
4-Hydroxybutanoic acid	HMDB0000549	1.16	1.36E-02	7.30E-03	0.57
L-Isoleucine	HMDB0000172	1.31	1.48E-02	7.73E-03	0.61
Glycerol	HMDB0000131	1.15	1.76E-02	8.61E-03	0.59
Stearic acid	HMDB0000827	1.08	2.81E-02	1.15E-02	0.44
Glycolic acid	HMDB0000115	1.11	2.96E-02	1.19E-02	0.53
Citric acid	HMDB0000094	1.07	3.05E-02	1.21E-02	0.56
Urea	HMDB0000294	1.12	3.52E-02	1.31E-02	0.61
L-Leucine	HMDB0000687	1.14	5.98E-02	1.79E-02	0.66
Liver					
(R)-3-Hydroxybutyric acid	HMDB0000011	1.82	1.19E-04	3.18E-03	0.40
Lactic Acid	HMDB0000190	1.69	7.27E-04	4.65E-03	0.44
Mesitylene	HMDB0041924	1.65	1.18E-03	5.28E-03	0.45
Dodecane	HMDB0031444	1.46	7.45E-03	2.29E-02	0.43
Glycolic acid	HMDB0000115	1.34	1.69E-02	3.98E-02	0.53
Cholesterol	HMDB0000067	1.33	1.83E-02	4.16E-02	0.57
Glycerol	HMDB0000131	1.32	1.85E-02	4.19E-02	0.61
Spleen					
Glycerol monostearate	HMDB0011535	1.65	9.92E-05	3.30E-03	0.63
1-Monopalmitin	HMDB0011564	1.65	1.16E-04	3.30E-03	0.51
Glycerol	HMDB0000131	1.53	1.30E-03	5.73E-03	0.62
Lactic Acid	HMDB0000190	1.49	2.00E-03	6.14E-03	0.59
Malic acid	HMDB0000744	1.42	2.84E-03	7.71E-03	0.51
1-Octadecanol	HMDB0002350	1.38	4.21E-03	1.04E-02	0.88
Stearic acid	HMDB0000827	1.40	6.06E-03	1.36E-02	0.55
L-5-Oxoproline	HMDB0000267	1.37	8.30E-03	1.66E-02	0.75
Cholesterol	HMDB0000067	1.42	8.71E-03	1.71E-02	0.58
Uridine	HMDB0000296	1.34	1.10E-02	1.95E-02	0.63
L-Proline	HMDB0000162	1.28	1.95E-02	2.99E-02	0.61
Tyrosine	HMDB0000158	1.18	2.15E-02	3.20E-02	0.77
Xylitol	HMDB0002917	1.17	2.82E-02	3.79E-02	0.54
Palmitic Acid	HMDB0000220	1.16	3.01E-02	3.94E-02	0.59
Myristic acid	HMDB0000806	1.12	3.16E-02	4.05E-02	0.69
Pentadecane	HMDB0059886	1.21	3.43E-02	4.24E-02	0.64
Kidney					
Butanedioic acid	HMDB0000254	1.53	3.83E-04	2.61E-03	2.93
Doconexent	HMDB0002183	1.51	5.14E-04	2.85E-03	2.84
L-5-Oxoproline	HMDB0000267	1.50	5.62E-04	2.92E-03	2.83
Decane	HMDB0031450	1.58	1.13E-03	3.99E-03	2.59
Cholesterol	HMDB0000067	1.52	1.50E-03	4.37E-03	2.41
Bisphenol A	HMDB0032133	1.42	1.63E-03	4.48E-03	2.45
1-Hexadecanol	HMDB0003424	1.43	1.92E-03	4.68E-03	2.00
Stearic acid	HMDB0000827	1.41	2.14E-03	4.80E-03	2.20
D-Gluconic acid	HMDB0000625	1.38	2.62E-03	5.18E-03	2.28
Malic acid	HMDB0000744	1.37	2.97E-03	5.41E-03	2.44
Palmitic Acid	HMDB0000220	1.46	3.43E-03	5.67E-03	2.14
Myo-Inositol	HMDB0000211	1.42	4.93E-03	6.91E-03	1.53
Glycerol	HMDB0000131	1.37	7.38E-03	8.71E-03	1.65
L-Threonine	HMDB0000167	1.26	7.95E-03	9.08E-03	1.81
L-Serine	HMDB0000187	1.25	8.65E-03	9.50E-03	2.40
Urea	HMDB0000294	1.21	1.28E-02	1.18E-02	4.66
L-Valine	HMDB0000883	1.19	1.45E-02	1.25E-02	2.24
L-Leucine	HMDB0000687	1.18	1.56E-02	1.30E-02	2.17
Niacinamide	HMDB0001406	1.16	1.67E-02	1.34E-02	2.97
L-Aspartic acid	HMDB0000191	1.16	1.68E-02	1.34E-02	2.49
Scyllo-Inositol	HMDB0006088	1.16	1.84E-02	1.42E-02	1.43
Arachidonic acid	HMDB0001043	1.12	2.67E-02	1.76E-02	2.06
Adonitol	HMDB0000508	1.10	2.88E-02	1.83E-02	2.09
D-(−)-Lactic acid	HMDB0001311	1.01	4.75E-02	2.63E-02	1.35
Dodecane	HMDB0031444	1.01	5.53E-02	2.91E-02	1.31
1-Octadecanol	HMDB0002350	1.05	5.80E-02	3.00E-02	1.62

### 3.5 Analysis of metabolic pathways

**FIGURE 5:**
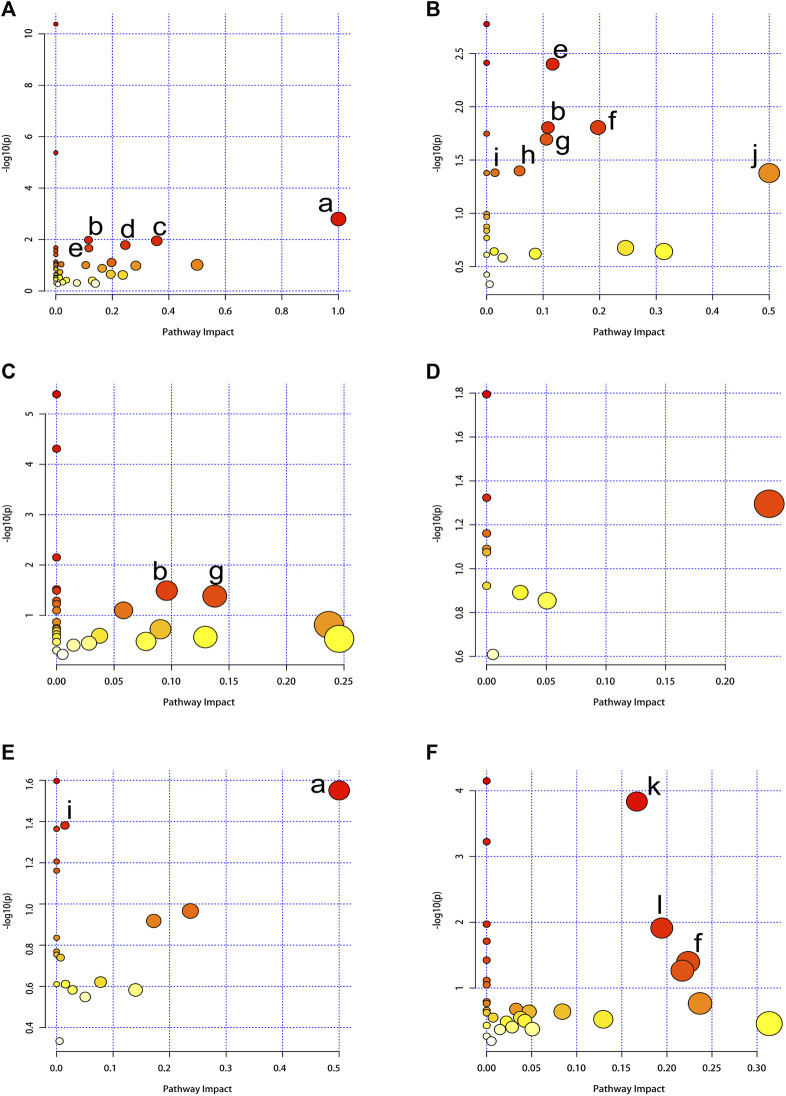
Summary of pathway analysis using MetaboAnalyst 5.0. The node color is based on the *p*-values (y-axis) and the node radius represents the pathway impact values (x-axis). **(A)** BAT, **(B)** WAT, **(C)** serum, **(D)** liver, **(E)** spleen, **(F)** kidney. (a) Phenylalanine, tyrosine and tryptophan biosynthesis. (b) Glutathione metabolism. (c) Phenylalanine metabolism. (d) Glycine, serine and threonine metabolism. (e) Arginine biosynthesis. (f) Alanine, aspartate and glutamate metabolism. (g) Glyoxylate and dicarboxylate metabolism. (h) Primary bile acid biosynthesis. (i) Fatty acid biosynthesis. (j) D-Glutamine and D-glutamate metabolism. (k) Aminoacyl-tRNA biosynthesis. (l) Nicotinate and nicotinamide metabolism.

**FIGURE 6:**
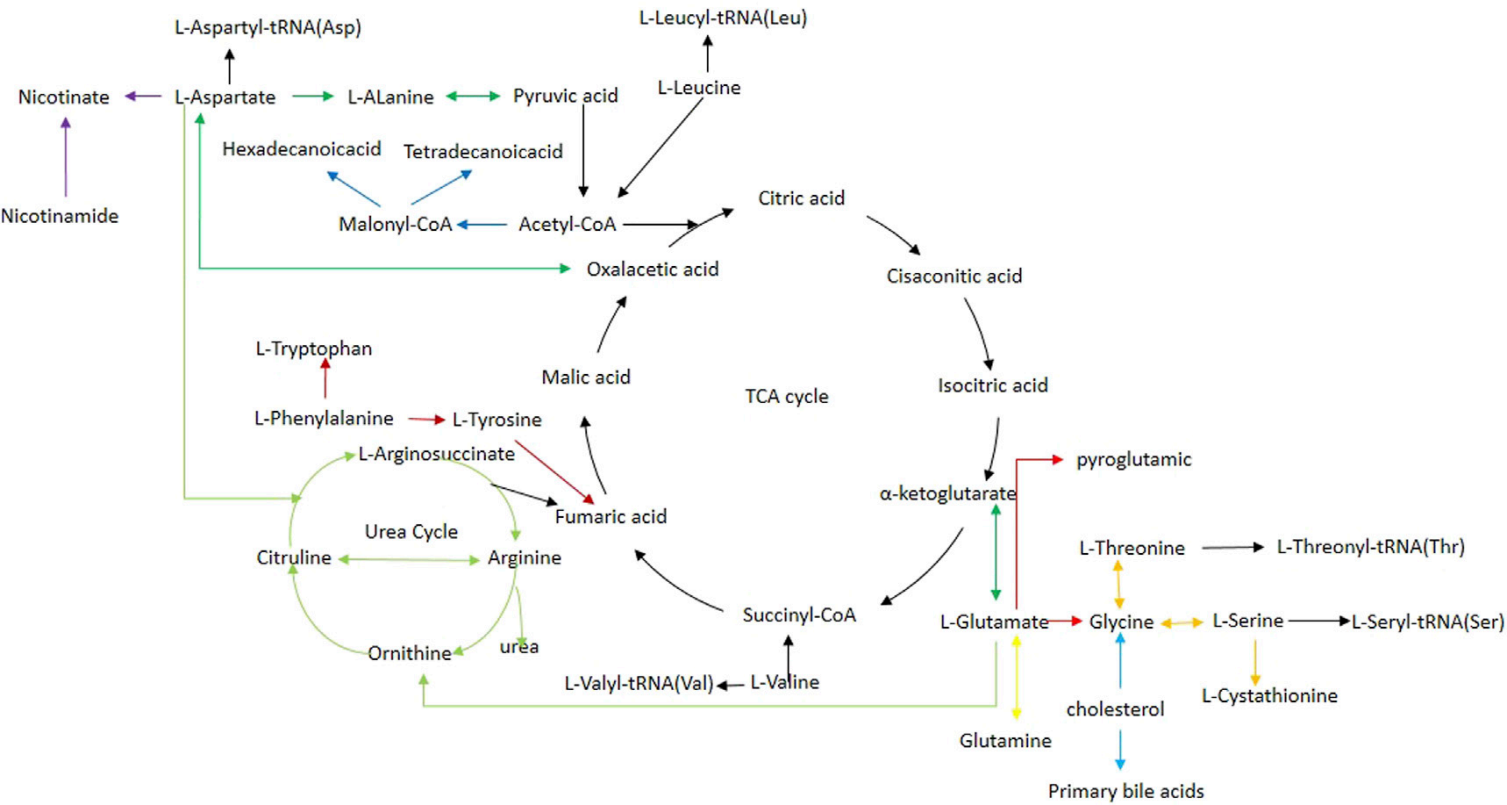
Schematic diagram of related metabolic pathways affected by CE processing in the main tissues. Black arrows indicate energy metabolism pathways, including the tricarboxylic acid cycle. Deep red arrows indicate the phenylalanine, tyrosine, and tryptophan biosynthesis. Red arrows indicate the glutathione metabolism pathway. Orange arrows indicate the glycine, serine, and threonine metabolism. Light green arrows indicate the arginine biosynthesis. Light blue arrows indicate the primary bile acid biosynthesis. Blue arrows indicate the fatty acid biosynthesis. Yellow arrows indicate the D-Glutamine and D-glutamate metabolism. Purple arrows indicate nicotinate and nicotinamide metabolism.

In our study, we employed MetaboAnalyst 5.0 to investigate the metabolic pathways associated with the specific metabolites identified through a comparison between the CE and control groups. Through our analysis, we identified 12 metabolic pathways that exhibited significant differences (with *p*-values adjusted <0.05 and impact values >0). In BAT, these pathways included phenylalanine, tyrosine and tryptophan biosynthesis; glutathione metabolism; phenylalanine metabolism; glycine, serine and threonine metabolism; as well as arginine biosynthesis. In WAT, these pathways included arginine biosynthesis; glutathione metabolism; alanine, aspartate and glutamate metabolism; glyoxylate and dicarboxylate metabolism; primary bile acid biosynthesis; fatty acid biosynthesis; as well as D-Glutamine and D-glutamate metabolism. In the serum, these pathways included glutathione metabolism; glyoxylate and dicarboxylate metabolism. In the spleen, these pathways included phenylalanine, tyrosine and tryptophan biosynthesis; along with fatty acid biosynthesis. In the kidney, these pathways included aminoacyl-tRNA biosynthesis; nicotinate and nicotinamide metabolism; as well as alanine, aspartate and glutamate metabolism. The specifics of the metabolic pathway analysis were depicted in Table 2; Figure 5. A summary of metabolic pathways was presented in Figure 6.

**TABLE 2:** Details of differential metabolic pathways in various tissues.

Tissue	Pathway name	Match status	Hits	P	P adjusted	Impact
BAT	Phenylalanine, tyrosine and tryptophan biosynthesis	2/4	L-Phenylalanine; L-Tyrosine	1.59E-03	1.17E-03	1.00E+00
	Glutathione metabolism	3/28	Glycine; L-Glutamate; 5-Oxoproline;	1.04E-02	4.42E-03	1.15E-01
	Phenylalanine metabolism	2/10	L-Phenylalanine; L-Tyrosine	1.12E-02	4.59E-03	3.57E-01
	Glycine, serine and threonine metabolism	3/33	L-Cystathionine; Glycine; L-Threonine;	1.64E-02	5.41E-03	2.46E-01
	Arginine biosynthesis	2/14	L-Glutamate; Urea	2.18E-02	5.99E-03	1.17E-01
WAT	Arginine biosynthesis	2/14	L-Glutamate; Urea	3.98E-03	2.47E-03	1.17E-01
	Glutathione metabolism	2/28	Glycine; L-Glutamate	1.57E-02	4.97E-03	1.08E-01
	Alanine, aspartate and glutamate metabolism	2/28	L-Alanine; L-Glutamate	1.57E-02	4.97E-03	1.97E-01
	Glyoxylate and dicarboxylate metabolism	2/32	Glycine; L-Glutamate;	2.02E-02	5.38E-03	1.06E-01
	Primary bile acid biosynthesis	2/46	Cholesterol; Glycine	4.00E-02	7.00E-03	5.82E-02
	Fatty acid biosynthesis	2/47	Hexadecanoic acid; Tetradecanoic acid	4.16E-02	7.08E-03	1.47E-02
	D-Glutamine and D-glutamate metabolism	1/6	L-Glutamate	4.19E-02	7.10E-03	5.00E-01
Serum	Glutathione metabolism	2/28	Glycine; 5-Oxoproline	3.23E-02	1.33E-02	9.58E-02
	Glyoxylate and dicarboxylate metabolism	2/32	Citrate; Glycine	4.14E-02	1.46E-02	1.38E-01
spleen	Phenylalanine, tyrosine and tryptophan biosynthesis	1/4	L-Tyrosine	2.81E-02	2.01E-02	5.00E-01
	Fatty acid biosynthesis	2/47	Hexadecanoic acid; Tetradecanoic acid	4.16E-02	2.01E-02	1.47E-02
kidney	Aminoacyl-tRNA biosynthesis	5/48	L-Aspartate; L-Serine; L-Valine; L-Leucine; L-Threonine	1.46E-04	2.03E-04	1.67E-01
	Nicotinate and nicotinamide metabolism	2/15	L-Aspartate; Nicotinamide	1.22E-02	6.79E-03	1.94E-01
	Alanine, aspartate and glutamate metabolism	2/28	L-Aspartate; Succinate	4.03E-02	1.40E-02	2.24E-01

## 4 Discussion

CE has significant effects on body metabolism. When exposed to cold temperatures, the body initiates a series of physiological responses to maintain core body temperature. These responses primarily involve increased energy expenditure to generate heat and altered metabolic processes.

In our study, we observed a significant increase in food intake in the CE group (*p* < 0.0001, Figure 1A); however, body weight was decreased (*p* < 0.05, Figure 1B). These findings were consistent with a previous report (Yang et al., 2017). One possible explanation for these results is that the cold-exposed mice needed to expend a significant amount of energy to generate heat and maintain their body temperature. The increase in energy consumption can potentially lead to higher food intake and a decrease in body weight.

Through GC-MS identification and statistical analysis, 32, 17, 21, 7, 16, and 26 differential metabolites were respectively identified in BAT, WAT, serum, liver, spleen and kidney after exposure to cold. These discriminant metabolites were involved in 12 pathways, mainly pertaining to amino acid, fatty acid, and energy metabolism.

Based on the altered metabolites and the associated differential metabolic pathways, it is evident that BAT was the most severely affected tissue. CE was also associated with varying degrees of metabolic effects in WAT, serum, liver, spleen and kidney. The metabolic effects in different tissues were discussed separately below.

### 4.1 BAT metabolism analysis

Branched chain amino acids (BCAAs), including leucine, isoleucine and valine, are essential amino acids. BCAAs can be oxidized to provide energy for cells (Sivanand and Vander Heiden, 2020). They are commonly converted into branched-chain alpha-ketoacids (BCKAs) through the catalytic action of branched-chain aminotransferases (BCATs) (Ichihara and Koyama, 1966). Subsequently, the branched-chain alpha-ketoacid dehydrogenase complex further converts the BCKAs into branched-chain acyl-CoA derivatives, which can then be metabolized into either acetyl-CoA or succinyl-CoA (Johnson and Connelly, 1972; Neinast et al., 2019). These metabolites eventually enter the tricarboxylic acid (TCA) cycle. In our study, we found that CE significantly increased the content of BCAAs in BAT. These findings were consistent with previous studies (Lu et al., 2017; Okamatsu-Ogura et al., 2020). According to Yoneshiro et al. (Yoneshiro et al., 2019), the higher BCAAs content in BAT is attributed to increased uptake from circulation. Indeed, previous studies have reported that the activity of BCATs increases following CE in rats (López-Soriano and Alemany, 1987). Consistent with this, more than 60% of genes encoding BCAAs catabolic enzymes, including the gene for the rate-limiting enzyme BCAT2, were more highly expressed in brown adipocytes relative to white adipocytes (Yoneshiro et al., 2019). Taken together, these data indicated that *in vivo*, BCAAs are used as significant energy sources for thermogenesis in BAT during CE. In our study, we observed a significant decrease in serum levels of BCAAs after exposure to cold, which was consistent with a previous human study (Kovaničová et al., 2021). Notably, elevated circulating BCAAs levels are closely linked to insulin resistance, obesity, and type 2 diabetes (Huffman et al., 2009; Newgard et al., 2009; Wang et al., 2011). CE enhances BCAAs clearance from the bloodstream by significantly increasing mitochondrial BCAAs uptake and oxidation in BAT (Yoneshiro et al., 2019). This process depends on the presence of SLC25A44, a mitochondrial BCAAs transporter in brown adipocytes, which might contribute to improved metabolic health (Yoneshiro et al., 2019). However, it remains unclear whether CE promotes the expression of *SLC25A44*, thereby enhancing BCAAs uptake. Further research is required.

Our study revealed a significant increase in glutamate levels in BAT following cold exposure, which aligns with previous research findings (Okamatsu-Ogura et al., 2020). A human study utilizing microdialysis demonstrated that CE increased glutamate uptake specifically by BAT (Weir et al., 2018), providing a potential explanation for the observed elevation in glutamate levels. Furthermore, exposure to cold in BAT resulted in an elevation of the expression of genes encoding glutamate dehydrogenase (GLUD1), an enzyme responsible for catalyzing the production of α-ketoglutaric acid from glutamate (Okamatsu-Ogura et al., 2020). These findings collectively suggest that glutamate serves as an important energy substrate for BAT thermogenesis *in vivo*. Additionally, we observed an increase in glycine content and an enhanced activity of the glutathione metabolism pathway in BAT after exposure to cold. CE triggers adaptive responses in the body, including the production of reactive oxygen species (ROS) as byproducts of increased metabolism and thermogenesis (Wang et al., 2015; Chouchani et al., 2016; Lettieri-Barbato, 2019). Uncontrolled ROS accumulation can lead to cellular oxidative damage. However, glutathione plays a crucial role as an antioxidant by scavenging ROS and protecting cells from oxidative stress. The observed enhancement in the glutathione metabolism pathway in BAT indicates an adaptive response to counteract the increased ROS generation during CE (Mory et al., 1983).

Lactic acid is produced through anaerobic glucose metabolism. Glucose transporters (GLUTs) play a vital role in cellular glucose uptake. In humans, there are a total of 14 different types of GLUTs (Thorens and Mueckler, 2010), with GLUT1 and GLUT4 expressed in BAT (James et al., 1988; James et al., 1989; Pessin and Bell, 1992). CE promotes a significant increase in the expression of GLUT1 and GLUT4 in mouse BAT (Daikoku et al., 2000; Yu et al., 2002). In line with this, studies using 18F-fluorodeoxyglucose-positron emission tomography/computed tomography (18F-FDG-PET/CT) have shown that CE enhances glucose uptake in the BAT of healthy humans (van Marken Lichtenbelt et al., 2009; Virtanen et al., 2009; Ouellet et al., 2012; Lee et al., 2016), *In vivo* [U-^13^C]glucose tracing experiments have also demonstrated that chronic cold exposure significantly increases glucose oxidation in BAT by promoting glucose flux into the mitochondrial TCA cycle (Wang et al., 2020). Notably, genes associated with glucose uptake and glycolysis were remarkably unregulated in cold-activated BAT (Hao et al., 2015). Taken together, these data indicate that CE enhances glucose uptake and oxidation. As we know, lactate dehydrogenases (LDH) play a crucial role in catalyzing the reversible conversion of pyruvate to lactate. It was observed that exposure to cold significantly induced the expression of *Ldha and Ldhb* mRNAs, as well as the LDHA protein in BAT (Hao et al., 2015). So, it is not surprising that lactic acid levels were significantly increased upon CE in BAT in our study. Previously considered a metabolic waste product, lactic acid is now recognized as a crucial metabolic fuel in BAT (Hui et al., 2017; Rabinowitz and Enerbäck, 2020). Recent studies have demonstrated that carbon derived from lactic acid can be utilized by brown adipocytes to synthesize fatty acids (Saggerson et al., 1988). As a result, lactic acid may play a role in swiftly replenishing BAT triglycerides following CE (Carpentier et al., 2023).

### 4.2 Serum metabolism analysis

Our study revealed that CE led to a decrease in the levels of fatty acids, specifically palmitic acid and stearic acid, as well as cholesterol in the serum of mice. CE induces the sympathetic nerves to release norepinephrine, which subsequently binds to β3-adrenoceptors (β3-ARs) found on the surface of adipocytes. This binding activates the cAMP–protein kinase A (PKA) signaling pathway, ultimately promoting intracellular TAG lipolysis (Cannon and Nedergaard, 2004; Zechner et al., 2012; Caron et al., 2018). When lipolysis occurs in WAT, it releases a significant amount of fatty acids into the bloodstream, which serve as the main energy substrate for BAT thermogenesis (Wang et al., 2021; Carpentier et al., 2023). Activated brown/beige adipocytes exhibit enhanced fatty acid uptake, as evidenced by 18F-fluoro-thiaheptadecanoic acid-positron emission tomography/computed tomography (18F-FTHA-PET/CT) imaging (Ouellet et al., 2012; Blondin et al., 2015). Moreover, CE causes a selective increase in lipoprotein lipase (LPL) expression in BAT (Chondronikola et al., 2016), while reducing the expression of angiopoietin-like 4 (ANGPTL4), an inhibitor of LPL activity (Dijk et al., 2015). As a result, activated BAT can effectively eliminate the majority of circulating triglyceride-rich lipoprotein (TRL) lipids (Bartelt et al., 2011; Berbée et al., 2015; Khedoe et al., 2015). Subsequently, fatty acids are transported into the mitochondria of brown fat cells via carnitine acyltransferases (CPTs), where they undergo β-oxidation and participate in the TCA cycle. Earlier research has demonstrated that CE can upregulate mRNA expression of *cpt* (Yu et al., 2002), thereby stimulating the β-oxidation of fatty acids in BAT of mice. Cold-activated BAT in mice holds promise for alleviating hyperlipidemia, providing benefits in conditions such as obesity or genetic hypertriglyceridemia (Bartelt et al., 2011; Berbée et al., 2015). Moreover, this activation indirectly reduces hypercholesterolemia by facilitating an increased hepatic uptake of cholesterol-enriched lipoprotein remnants, thereby offering protection against the development of atherosclerosis (Berbée et al., 2015; Bartelt et al., 2017).

### 4.3 Liver metabolism analysis

The liver’s role in maintaining cholesterol balance is pivotal due to its ability to uptake, synthesize, convert cholesterol into bile acids, and excrete cholesterol within very low-density lipoprotein (VLDL) particles. Our study observed a substantial reduction in liver cholesterol levels following exposure to cold conditions. This phenomenon could be attributed to the cold-induced conversion of cholesterol into bile acids in the liver (Worthmann et al., 2017). Specifically, the researchers found that the levels of most bile acid species in the liver were notably higher in mice housed in cold conditions compared to the control group in warm conditions. Additionally, the study revealed a significant upregulation in the expression of the important gene *Cyp7b*, which is involved in alternative bile acid synthesis pathways, in response to CE (Worthmann et al., 2017).

### 4.4 Spleen metabolism analysis

The spleen plays a crucial role as a lymphatic and immune organ in the human body. Our research has revealed a decrease in the levels of lactic acid and malic acid within the spleen during CE, indicating a weakening of anaerobic respiration and TCA cycle processes. Furthermore, a transcriptomic study has indicated that gene sets associated with biological processes, including the innate immune response in mucosa, neutrophil-mediated killing of symbiont cells, inflammatory response, and various other responsive systems, were downregulated in the spleen (Hadadi et al., 2022). This phenomenon can be attributed to the spleen’s primary immunological functions, a biological process that might become less effective during cold conditions. This aligns with the concept that maintaining immune functions requires significant energy expenditure, potentially competing with other energy-demanding functions, including thermogenesis (Spiljar et al., 2021). Furthermore, the process of triglyceride catabolism was found to be subdued in the spleen during cold exposure (Hadadi et al., 2022). It is not unexpected that the levels of glycerol and fatty acids (such as palmitic acid, stearic acid, myristic acid, glycerol monostearate, and 1-Monopalmitin) showed a decrease within the spleen according to our study. To some extent, this could suggest a redistribution of metabolic energy towards the tissues essential for responding to cold, as a consequence of prioritizing other maintenance-related biological processes.

### 4.5 Kidney metabolism analysis

The kidneys play a vital role in regulating various essential physiological functions. They are responsible for producing urine, removing metabolic toxins, and maintaining the balance of water, electrolytes, and acid-base levels (Imenez Silva and Mohebbi, 2022). Consequently, the kidneys are organs with high metabolic activity (Clark and Parikh, 2020). Research has indicated that the kidneys have the second highest concentration of mitochondria per weight, surpassed only by the heart (Pagliarini et al., 2008). To meet their energy demands, the kidneys metabolize significant amounts of nutrients, such as fatty acids, glucose, and amino acids.

Nicotinamide (NAM), a member of the vitamin B3 group, has the capacity to generate nicotinamide adenine dinucleotide (NAD^+^). NAD^+^ plays a crucial role as an electron acceptor in several catabolic processes, including glycolysis, the TCA cycle, and fatty acid β-oxidation (FAO). Subsequently, the acquired electrons are transferred to oxygen through the mitochondrial respiratory chain, leading to the conversion of ADP into ATP via phosphorylation (Fontecha-Barriuso et al., 2021). In our research, following CE, we observed a significant increase in NAM levels and a notable enhancement in the pathway of nicotinate and NAM metabolism. Based on this, we hypothesized that CE may enhance renal energy metabolism. However, a recent time-series study conducted in South Korea identified a correlation between CE and hospital admissions and deaths associated with acute kidney injury (AKI) (Kim et al., 2023). These conflicting results could be attributed to various factors, such as the duration and intensity of CE, the individual’s health condition, and their capacity to adapt to the cold. Consequently, further research is necessary to investigate the impact of CE on renal metabolism in both animal models and clinical settings.

### 4.6 Interorgan communication in response to CE

The process of cold-induced thermogenesis is intricately tied to a well-coordinated metabolic adjustment program spanning multiple tissues. This program functions to uphold the equilibrium of fuel and energy within the body. Cold stimuli trigger the initiation of lipolysis within WAT, leading to the liberation of free fatty acids (FFAs). These FFAs either act as direct substrates for BAT-mediated thermogenesis or journey to the liver. In the liver, these FFAs undergo a transformation into acylcarnitines, facilitated by an increase in the expression of the *CPT1* gene, brought about by the activation of HNF4a (Simcox et al., 2017). The acylcarnitines produced in the liver are predominantly taken up by BAT (Simcox et al., 2017), where they contribute significantly to thermogenic processes. Traditionally, bile acids, generated solely by the liver through the conversion of cholesterol, can follow either the classical pathway or the alternative pathway. Cold exposure particularly triggers the activation of the alternative pathway, leading to a substantial rise in bile acids expelled through feces (Worthmann et al., 2017). This surge is accompanied by a distinct transformation in the composition of the gut microbiome (Worthmann et al., 2017). This altered configuration of the microbiome has the potential to result in the creation of microbial metabolites possessing inherent thermogenic properties (Worthmann et al., 2017).

While our study provided valuable insights, it is crucial to acknowledge its limitations. In order to achieve a comprehensive understanding of the metabolic alterations induced by CE, it is imperative to explore its effects on other tissues, such as the heart, lung, cortex, hippocampus, stomach, pancreas, skin, and bone. However, obtaining samples from multiple tissues is challenging and time-consuming, as it involves the collection, rinsing, and freezing of multiple tissues in liquid nitrogen. Additionally, many metabolites have a short turnover time, which could potentially impact the accuracy of metabolite measurements. Furthermore, it’s worth noting that the use of anesthesia during the sampling procedure can induce metabolic changes in tissues, potentially influencing the results. Another factor to consider is that food intake was not normalized between groups, which could contribute to the observed differences in metabolites (Jang et al., 2018).

## 5 Conclusion

Metabolic changes induced by CE in multiple tissues in mice were analyzed using GC-MS, providing a deeply understanding of the effects of CE. We observed that CE altered the levels of various metabolites across multiple tissues, with notable impacts on amino acids metabolism, fatty acids metabolism and energy metabolism. Our findings provide systematic insights into the metabolic impacts induced by CE and may also present new ideas for treating metabolic diseases.

## Data Availability

The original contributions presented in the study are included in the article/Supplementary Material, further inquiries can be directed to the corresponding authors.
